# Supplementary material for: Bite marks and predation of fossil jawless fish during the rise of jawed vertebrates
Source: Proc Biol Sci. 2019 Dec 18;286(1917):20191596. doi: 10.1098/rspb.2019.1596 (PMC6939932; doi:10.1098/rspb.2019.1596)
Supplement: Supplementary Figures [file rspb20191596supp1.docx]

**Supplementary Material for: “Bite marks and predation of fossil jawless fish during the rise of jawed vertebrates”**

**Randle and Sansom**


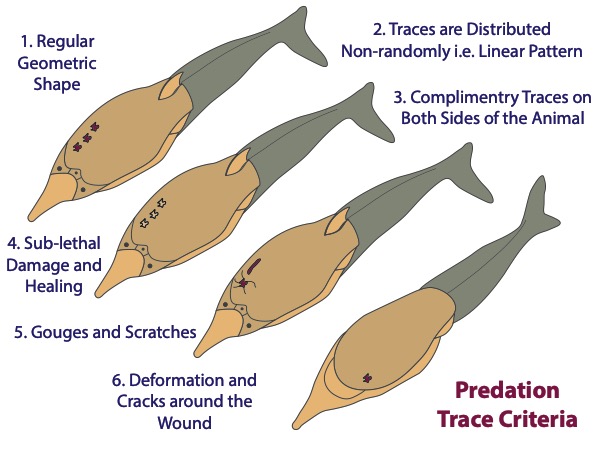


**Supplementary Figure 1**. Criteria for identifying predation traces in jawless heterostracan fossils.


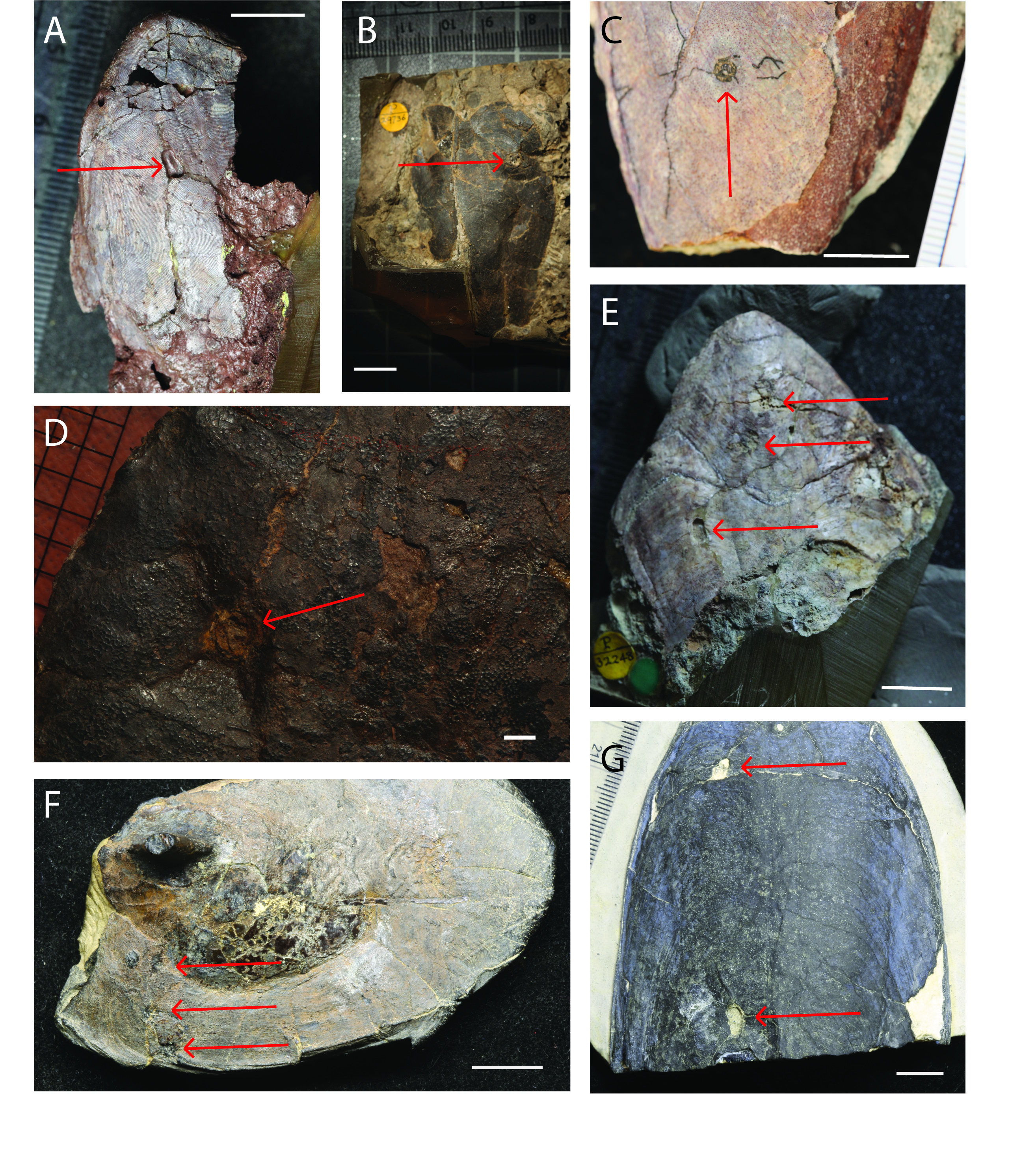


**Supplementary Figure 2.** Specimens displaying predation traces (identified by red arrow), scale = 10mm. A. *Protopteraspis gosselletti* (P.34120, NHM). B. *Rhinopteraspis crouchi* (P.29736, NHM). C. *Torpedaspis elongate* (NMC.12633). D. *Pelurgaspis macrorhyncha* (PIN.1489/3 Paleontological Institute). E. *Loricopteraspis dairydinglensis (*P.32248, NHM). F. *Eucyclaspis erroli* (PF.3814, Field Museum). G. *Blieckaspis priscillae* (PF.867, Field Museum).

**Supplementary Figure 3** Power Analysis. Power of correlation analyses at different effect sizes (r, in this case Spearman’s rho) and different numbers of observations (n) at 0.05 significance level. The correlation values found in the tests for increasing prevalence through time are also depicted (r=0.83, 0.74).
